# 2-Amino-4′-bromo-2′,5-dioxo-4*H*,5*H*-pyrano[3,2-*c*]chromene-4-spiro-3′(2′*H*)-1′*H*-indole-3-carbonitrile *N*,*N*-dimethyl­formamide solvate

**DOI:** 10.1107/S1600536808015626

**Published:** 2008-05-30

**Authors:** Song-Lei Zhu

**Affiliations:** a Department of Chemistry, Xuzhou Medical College, Xuzhou 221002, People’s Republic of China

## Abstract

In the mol­ecule of the title compound, C_20_H_10_BrN_3_O_4_·C_3_H_7_NO, the spiro pyran ring adopts a twist conformation. The indole and coumarin ring systems are each nearly planar, and are oriented at a dihedral angle of 79.29 (3)°. In the crystal structure, inter­molecular N—H⋯O, N—H⋯N, C—H⋯O and C—H⋯N hydrogen bonds link the mol­ecules.

## Related literature

For general background, see: da Silva *et al.* (2001 ▶); Joshi & Chand (1982 ▶); Abdel-Rahman *et al.* (2004 ▶); Zhu *et al.* (2007 ▶). For ring-puckering parameters, see: Cremer & Pople (1975 ▶).
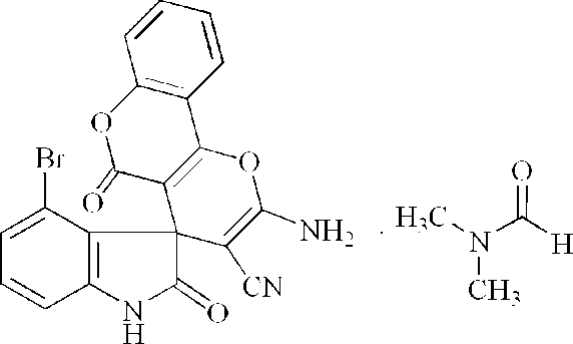


## Experimental

### 

#### Crystal data

C_20_H_10_BrN_3_O_4_·C_3_H_7_NO
*M* *_r_* = 509.32
Monoclinic, 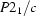

*a* = 17.004 (3) Å
*b* = 9.0452 (15) Å
*c* = 14.415 (3) Å
β = 108.340 (3)°
*V* = 2104.5 (7) Å^3^
*Z* = 4
Mo *K*α radiation
μ = 2.00 mm^−1^
*T* = 153 (2) K
0.45 × 0.30 × 0.20 mm

#### Data collection

Rigaku Mercury diffractometer
Absorption correction: multi-scan (Jacobson, 1998 ▶) *T*_min_ = 0.434, *T*_max_ = 0.670
19919 measured reflections
3847 independent reflections
3597 reflections with *I* > 2σ(*I*)
*R*_int_ = 0.031

#### Refinement

*R*[*F* ^2^ > 2σ(*F* ^2^)] = 0.031
*wR*(*F* ^2^) = 0.067
*S* = 1.09
3847 reflections
309 parameters
H atoms treated by a mixture of independent and constrained refinement
Δρ_max_ = 0.56 e Å^−3^
Δρ_min_ = −0.38 e Å^−3^

### 

Data collection: *CrystalClear* (Rigaku/MSC, 2001 ▶); cell refinement: *CrystalClear*; data reduction: *CrystalStructure* (Rigaku/MSC, 2004 ▶); program(s) used to solve structure: *SHELXS97* (Sheldrick, 2008 ▶); program(s) used to refine structure: *SHELXL97* (Sheldrick, 2008 ▶); molecular graphics: *ORTEPII* (Johnson, 1976 ▶); software used to prepare material for publication: *SHELXL97*.

## Supplementary Material

Crystal structure: contains datablocks global, I. DOI: 10.1107/S1600536808015626/hk2467sup1.cif

Structure factors: contains datablocks I. DOI: 10.1107/S1600536808015626/hk2467Isup2.hkl

Additional supplementary materials:  crystallographic information; 3D view; checkCIF report

## Figures and Tables

**Table 1:** Hydrogen-bond geometry (Å, °)

*D*—H⋯*A*	*D*—H	H⋯*A*	*D*⋯*A*	*D*—H⋯*A*
N1—H1*A*⋯O2^i^	0.89 (3)	2.02 (3)	2.891 (2)	165 (2)
N1—H1*B*⋯N2^ii^	0.84 (3)	2.27 (3)	3.090 (3)	166 (2)
N3—H3⋯O5^iii^	0.88	1.93	2.785 (2)	163
C11—H11⋯O2^iv^	0.95	2.54	3.462 (3)	165
C19—H19⋯O4^i^	0.95	2.50	3.173 (3)	128
C22—H22*A*⋯N2^v^	0.98	2.48	3.443 (3)	166

Symmetry codes: (i) 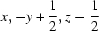; (ii) 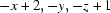; (iii) 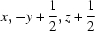; (iv) 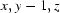; (v) 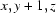.

**Fig. 1.**
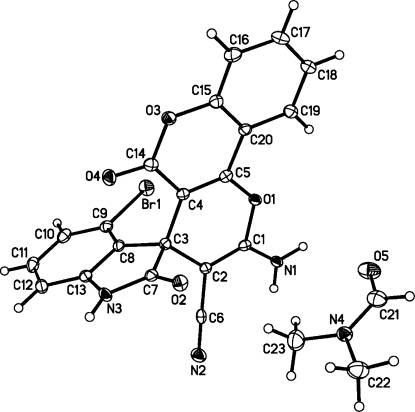
The molecular structure of the title molecule, with the atom-numbering scheme. Displacement ellipsoids are drawn at the 40% probability level.

**Fig. 2.**
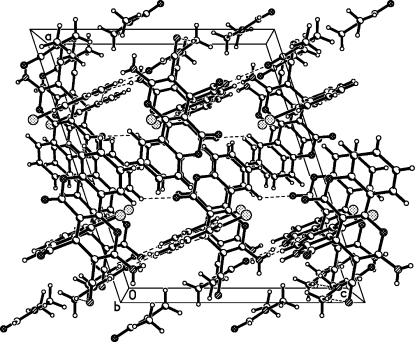
A packing diagram of (I). Hydrogen bonds are shown as dashed lines.

## supplementary crystallographic information

### Comment

The indole nucleus is the well known heterocycle (da-Silva *et al.*, 2001). Compounds carrying the indole moiety exhibit antibacterial and fungicidal activities (Joshi & Chand, 1982). Spirooxindole ring systems are found in a number of alkaloids like horsifiline, spirotryprostatin and elacomine (Abdel-Rahman *et al.*, 2004). As a part of our programme devoted to the preparation of heterocyclic compounds involving indole derivatives (Zhu *et al.*,  2007), we have synthesized a series of spirooxindoles *via* reactions of substituted isatins together with malononitrile and 4-hydroxycoumarin in water. We report herein the crystal structure of the title compound, (I).

In the molecule of (I), (Fig. 1), rings B (N3/C3/C7/C8/C13), C (C8-C13), D (O3/C4/C5/C14/C15/C20) and E (C15-C20) are, of course, planar. The dihedral angles between them are B/C = 1.51 (3)° and D/E = 4.24 (3)°. So, rings B, C and D,E are nearly coplanar. The coplanar ring systems are oriented at a dihedral angle of 79.29 (3)°. Ring A (O1/C1-C5) adopts twisted conformation, having total puckering amplitude, Q_T_, of 0.122 (3) Å (Cremer & Pople, 1975).

In the crystal structure, intermolecular N-H···O, N-H···N, C-H···O and C-H···N hydrogen bonds (Table 1) link the molecules (Fig. 2), in which they may be effective in the stabilization of the structure.

### Experimental

Compound (I) was prepared by the reaction of 4-bromoisatin (1 mmol), malononitrile (1 mmol) and 4-hydroxycoumarin (1 mmol) in water (5 ml). The reaction was catalyzed by TEBAC (triethylbenzylammonium chloride, 1 mmol). After stirring at 333 K for 5 h, the reaction mixture was cooled and washed with small amount of ethanol. The crude product was filtered and single crystals of the title compound were obtained from DMF solution by slow evaporation at room temperature (yield; 80%, m.p. > 573 K). Spectroscopic analysis: IR (KBr, n, cm^-1^): 3372, 3310, 3179, 2192, 1728, 1674, 1605, 1450, 1358, 1234, 1111, 1080, 972, 910, 872, 764, 578. ^1^H NMR (400 MHz, DMSO-d_6_): 10.99 (s, 1H, NH), 7.96 (d, 1H, J = 7.6 Hz, ArH), 7.84 (br s, 2H, NH2), 7.79 (t, 1H, J = 8.4 Hz, ArH), 7.53–7.59 (m, 2H, ArH), 7.21 (t, 1H, J = 8.0 Hz, ArH), 7.12 (d, 1H, J = 8.0 Hz, ArH), 6.91 (d, 1H, J = 7.6 Hz, ArH).

### Refinement

H atoms (for NH_2_) were located in a difference syntheses and refined [N-H = 0.89 (3) and 0.84 (3) Å; U_iso_(H) = 0.022 (7) and 0.018 (6) Å^2^]. The remaining H atoms were positioned geometrically, with N-H = 0.88 Å (for NH) and C-H = 0.95 and 0.98 Å for aromatic and methyl H and constrained to ride on their parent atoms with U_iso_(H) = xU_eq_(C,N), where x = 1.5 for methyl H and x = 1.2 for all other H atoms.

### Crystal data

**Table:**

C_20_H_10_BrN_3_O_4_·C_3_H_7_NO	*F*_000_ = 1032
*M**_r_* = 509.32	*D*_x_ = 1.607 Mg m^−^^3^
Monoclinic, *P*2_1_/*c*	Melting point > 573 K
Hall symbol: -P 2 y b c	Mo *K*α radiation λ = 0.71070 Å
*a* = 17.004 (3) Å	Cell parameters from 7931 reflections
*b* = 9.0452 (15) Å	θ = 3.2–25.3º
*c* = 14.415 (3) Å	µ = 2.00 mm^−^^1^
β = 108.340 (3)º	*T* = 153 (2) K
*V* = 2104.5 (7) Å^3^	Block, colorless
*Z* = 4	0.45 × 0.30 × 0.20 mm

### Data collection

**Table:**

Rigaku Mercury diffractometer	3847 independent reflections
Radiation source: fine-focus sealed tube	3597 reflections with *I* > 2σ(*I*)
Monochromator: graphite	*R*_int_ = 0.031
Detector resolution: 7.31 pixels mm^-1^	θ_max_ = 25.3º
*T* = 153(2) K	θ_min_ = 3.2º
ω scans	*h* = −17→20
Absorption correction: multi-scan(Jacobson, 1998)	*k* = −10→9
*T*_min_ = 0.434, *T*_max_ = 0.670	*l* = −17→17
19919 measured reflections	

### Refinement

**Table:**

Refinement on *F*^2^	Secondary atom site location: difference Fourier map
Least-squares matrix: full	Hydrogen site location: inferred from neighbouring sites
*R*[*F*^2^ > 2σ(*F*^2^)] = 0.031	H atoms treated by a mixture of independent and constrained refinement
*wR*(*F*^2^) = 0.067	*w* = 1/[σ^2^(*F*_o_^2^) + (0.0219*P*)^2^ + 2.2795*P*] where *P* = (*F*_o_^2^ + 2*F*_c_^2^)/3
*S* = 1.09	(Δ/σ)_max_ = 0.002
3847 reflections	Δρ_max_ = 0.56 e Å^−^^3^
309 parameters	Δρ_min_ = −0.38 e Å^−^^3^
Primary atom site location: structure-invariant direct methods	Extinction correction: none

### Special details

**Table:**

Geometry. All e.s.d.'s (except the e.s.d. in the dihedral angle between two l.s. planes) are estimated using the full covariance matrix. The cell e.s.d.'s are taken into account individually in the estimation of e.s.d.'s in distances, angles and torsion angles; correlations between e.s.d.'s in cell parameters are only used when they are defined by crystal symmetry. An approximate (isotropic) treatment of cell e.s.d.'s is used for estimating e.s.d.'s involving l.s. planes.
Refinement. Refinement of F^2^ against ALL reflections. The weighted R-factor wR and goodness of fit S are based on F^2^, conventional R-factors R are based on F, with F set to zero for negative F^2^. The threshold expression of F^2^ > 2sigma(F^2^) is used only for calculating R-factors(gt) etc. and is not relevant to the choice of reflections for refinement. R-factors based on F^2^ are statistically about twice as large as those based on F, and R- factors based on ALL data will be even larger.

### Fractional atomic coordinates and isotropic or equivalent isotropic displacement parameters (Å^2^)

**Table:**

	*x*	*y*	*z*	*U*_iso_*/*U*_eq_	
Br1	0.674546 (15)	−0.23487 (3)	0.370899 (16)	0.02528 (8)	
O1	0.72297 (9)	0.17427 (16)	0.38347 (10)	0.0165 (3)	
O2	0.80028 (9)	0.17370 (16)	0.69972 (11)	0.0195 (3)	
O3	0.54289 (9)	0.16195 (17)	0.51984 (11)	0.0205 (3)	
O4	0.61528 (9)	0.00093 (17)	0.62894 (11)	0.0218 (3)	
O5	0.86929 (11)	0.5130 (2)	0.42641 (12)	0.0350 (4)	
N1	0.84875 (12)	0.1416 (2)	0.37211 (14)	0.0165 (4)	
H1A	0.8332 (15)	0.211 (3)	0.3263 (19)	0.022 (7)*	
H1B	0.8976 (16)	0.110 (3)	0.3921 (17)	0.018 (6)*	
N2	0.97044 (12)	−0.0393 (2)	0.58997 (14)	0.0245 (4)	
N3	0.80382 (11)	−0.07714 (19)	0.72793 (13)	0.0170 (4)	
H3	0.8267	−0.0767	0.7918	0.020*	
N4	0.94175 (11)	0.5142 (2)	0.58794 (13)	0.0216 (4)	
C1	0.79982 (12)	0.1140 (2)	0.42627 (15)	0.0135 (4)	
C2	0.81869 (12)	0.0379 (2)	0.51188 (15)	0.0135 (4)	
C3	0.75548 (12)	−0.0029 (2)	0.56233 (15)	0.0132 (4)	
C4	0.67684 (12)	0.0815 (2)	0.51288 (15)	0.0135 (4)	
C5	0.66610 (13)	0.1649 (2)	0.43275 (15)	0.0143 (4)	
C6	0.90223 (13)	−0.0065 (2)	0.55498 (15)	0.0159 (4)	
C7	0.78839 (12)	0.0454 (2)	0.67200 (15)	0.0154 (4)	
C8	0.74743 (12)	−0.1686 (2)	0.57294 (15)	0.0140 (4)	
C9	0.71969 (13)	−0.2801 (2)	0.50567 (15)	0.0166 (4)	
C10	0.72305 (14)	−0.4273 (2)	0.53575 (17)	0.0206 (5)	
H10	0.7035	−0.5039	0.4891	0.025*	
C11	0.75512 (14)	−0.4604 (2)	0.63403 (18)	0.0229 (5)	
H11	0.7577	−0.5607	0.6543	0.027*	
C12	0.78381 (13)	−0.3505 (2)	0.70414 (17)	0.0199 (5)	
H12	0.8059	−0.3739	0.7716	0.024*	
C13	0.77895 (13)	−0.2058 (2)	0.67179 (15)	0.0153 (4)	
C14	0.61196 (13)	0.0753 (2)	0.55878 (15)	0.0168 (5)	
C15	0.53467 (13)	0.2528 (2)	0.44043 (16)	0.0178 (5)	
C16	0.46555 (14)	0.3429 (3)	0.41187 (18)	0.0247 (5)	
H16	0.4252	0.3389	0.4448	0.030*	
C17	0.45632 (14)	0.4387 (3)	0.33474 (18)	0.0264 (5)	
H17	0.4097	0.5026	0.3153	0.032*	
C18	0.51418 (14)	0.4430 (3)	0.28502 (17)	0.0240 (5)	
H18	0.5066	0.5087	0.2315	0.029*	
C19	0.58252 (13)	0.3522 (2)	0.31330 (16)	0.0195 (5)	
H19	0.6220	0.3550	0.2791	0.023*	
C20	0.59384 (13)	0.2557 (2)	0.39248 (15)	0.0156 (4)	
C21	0.90376 (15)	0.5775 (3)	0.50259 (18)	0.0290 (6)	
H21	0.9034	0.6825	0.5007	0.035*	
C22	0.98163 (16)	0.6027 (3)	0.67431 (19)	0.0330 (6)	
H22A	0.9725	0.7079	0.6581	0.050*	
H22B	1.0412	0.5821	0.6968	0.050*	
H22C	0.9580	0.5777	0.7262	0.050*	
C23	0.94561 (18)	0.3560 (3)	0.6002 (2)	0.0376 (7)	
H23A	0.9097	0.3257	0.6382	0.056*	
H23B	1.0028	0.3264	0.6347	0.056*	
H23C	0.9270	0.3082	0.5359	0.056*	

### Atomic displacement parameters (Å^2^)

**Table:**

	*U*^11^	*U*^22^	*U*^33^	*U*^12^	*U*^13^	*U*^23^
Br1	0.03215 (15)	0.02519 (14)	0.01580 (13)	−0.00378 (10)	0.00365 (10)	−0.00394 (9)
O1	0.0129 (7)	0.0220 (8)	0.0152 (8)	0.0032 (6)	0.0053 (6)	0.0050 (6)
O2	0.0243 (8)	0.0164 (8)	0.0170 (8)	−0.0025 (6)	0.0051 (7)	−0.0043 (6)
O3	0.0157 (8)	0.0256 (9)	0.0225 (8)	0.0047 (6)	0.0095 (7)	0.0063 (7)
O4	0.0234 (8)	0.0243 (9)	0.0204 (8)	0.0027 (7)	0.0110 (7)	0.0059 (7)
O5	0.0318 (10)	0.0512 (12)	0.0194 (9)	0.0038 (9)	0.0042 (8)	−0.0030 (8)
N1	0.0130 (10)	0.0197 (10)	0.0172 (10)	0.0038 (8)	0.0051 (8)	0.0054 (8)
N2	0.0193 (11)	0.0316 (12)	0.0235 (10)	0.0053 (9)	0.0079 (9)	0.0070 (9)
N3	0.0199 (10)	0.0187 (10)	0.0105 (9)	0.0003 (8)	0.0022 (7)	0.0023 (7)
N4	0.0205 (10)	0.0256 (11)	0.0180 (10)	−0.0003 (8)	0.0051 (8)	0.0019 (8)
C1	0.0119 (10)	0.0122 (10)	0.0160 (10)	−0.0005 (8)	0.0037 (8)	−0.0037 (8)
C2	0.0129 (10)	0.0137 (11)	0.0139 (10)	0.0000 (8)	0.0043 (8)	−0.0003 (8)
C3	0.0137 (10)	0.0142 (11)	0.0119 (10)	−0.0009 (8)	0.0045 (8)	−0.0006 (8)
C4	0.0131 (10)	0.0135 (10)	0.0133 (10)	−0.0008 (8)	0.0034 (8)	−0.0025 (8)
C5	0.0141 (10)	0.0153 (11)	0.0143 (10)	−0.0015 (8)	0.0055 (8)	−0.0040 (8)
C6	0.0216 (12)	0.0145 (11)	0.0140 (11)	−0.0012 (9)	0.0089 (9)	0.0020 (8)
C7	0.0122 (10)	0.0194 (12)	0.0151 (11)	0.0003 (8)	0.0051 (9)	0.0014 (9)
C8	0.0121 (10)	0.0137 (11)	0.0179 (11)	0.0012 (8)	0.0070 (9)	0.0016 (8)
C9	0.0137 (10)	0.0213 (11)	0.0158 (11)	0.0000 (9)	0.0063 (9)	−0.0006 (9)
C10	0.0195 (11)	0.0158 (11)	0.0290 (13)	−0.0023 (9)	0.0112 (10)	−0.0042 (9)
C11	0.0224 (12)	0.0145 (11)	0.0356 (14)	0.0031 (9)	0.0146 (11)	0.0060 (10)
C12	0.0191 (11)	0.0201 (12)	0.0222 (12)	0.0030 (9)	0.0090 (10)	0.0061 (9)
C13	0.0133 (10)	0.0172 (11)	0.0167 (11)	0.0013 (8)	0.0064 (9)	0.0009 (9)
C14	0.0161 (11)	0.0160 (11)	0.0172 (11)	0.0000 (9)	0.0036 (9)	−0.0026 (9)
C15	0.0163 (11)	0.0187 (11)	0.0175 (11)	−0.0012 (9)	0.0042 (9)	0.0002 (9)
C16	0.0181 (12)	0.0276 (13)	0.0312 (13)	0.0031 (10)	0.0117 (10)	0.0008 (10)
C17	0.0180 (12)	0.0272 (13)	0.0313 (13)	0.0085 (10)	0.0041 (10)	0.0050 (10)
C18	0.0186 (12)	0.0263 (13)	0.0237 (12)	0.0025 (10)	0.0016 (10)	0.0071 (10)
C19	0.0179 (11)	0.0212 (12)	0.0182 (11)	0.0009 (9)	0.0040 (9)	0.0015 (9)
C20	0.0131 (10)	0.0158 (11)	0.0159 (11)	−0.0019 (8)	0.0018 (9)	−0.0014 (8)
C21	0.0244 (13)	0.0374 (15)	0.0263 (14)	0.0049 (11)	0.0097 (11)	0.0008 (11)
C22	0.0299 (14)	0.0340 (15)	0.0299 (14)	0.0011 (11)	0.0017 (11)	−0.0052 (11)
C23	0.0446 (17)	0.0293 (15)	0.0357 (15)	−0.0074 (12)	0.0078 (13)	0.0020 (12)

### Geometric parameters (Å, °)

**Table:**

Br1—C9	1.895 (2)	C8—C9	1.376 (3)
O1—C1	1.370 (2)	C8—C13	1.396 (3)
O1—C5	1.371 (2)	C9—C10	1.395 (3)
O2—C7	1.223 (3)	C10—C11	1.381 (3)
O3—C14	1.376 (3)	C10—H10	0.9500
O3—C15	1.380 (3)	C11—C12	1.392 (3)
O4—C14	1.201 (3)	C11—H11	0.9500
O5—C21	1.219 (3)	C12—C13	1.383 (3)
N1—C1	1.331 (3)	C12—H12	0.9500
N1—H1A	0.89 (3)	C15—C16	1.382 (3)
N1—H1B	0.84 (3)	C15—C20	1.389 (3)
N2—C6	1.149 (3)	C16—C17	1.379 (3)
N3—C7	1.347 (3)	C16—H16	0.9500
N3—C13	1.404 (3)	C17—C18	1.388 (3)
N3—H3	0.8800	C17—H17	0.9500
N4—C21	1.326 (3)	C18—C19	1.376 (3)
N4—C23	1.441 (3)	C18—H18	0.9500
N4—C22	1.456 (3)	C19—C20	1.401 (3)
C1—C2	1.360 (3)	C19—H19	0.9500
C2—C6	1.418 (3)	C21—H21	0.9500
C2—C3	1.521 (3)	C22—H22A	0.9800
C3—C4	1.509 (3)	C22—H22B	0.9800
C3—C8	1.517 (3)	C22—H22C	0.9800
C3—C7	1.564 (3)	C23—H23A	0.9800
C4—C5	1.343 (3)	C23—H23B	0.9800
C4—C14	1.455 (3)	C23—H23C	0.9800
C5—C20	1.440 (3)		
			
C1—O1—C5	118.06 (16)	C12—C11—H11	119.2
C14—O3—C15	121.88 (16)	C13—C12—C11	117.5 (2)
C1—N1—H1A	118.2 (16)	C13—C12—H12	121.3
C1—N1—H1B	118.0 (16)	C11—C12—H12	121.3
H1A—N1—H1B	122 (2)	C12—C13—C8	122.3 (2)
C7—N3—C13	111.74 (17)	C12—C13—N3	127.9 (2)
C7—N3—H3	124.1	C8—C13—N3	109.74 (18)
C13—N3—H3	124.1	O4—C14—O3	118.06 (19)
C21—N4—C23	122.2 (2)	O4—C14—C4	124.3 (2)
C21—N4—C22	121.1 (2)	O3—C14—C4	117.68 (18)
C23—N4—C22	116.8 (2)	O3—C15—C16	116.93 (19)
N1—C1—C2	127.9 (2)	O3—C15—C20	121.61 (19)
N1—C1—O1	110.17 (18)	C16—C15—C20	121.4 (2)
C2—C1—O1	121.94 (18)	C17—C16—C15	118.8 (2)
C1—C2—C6	117.31 (18)	C17—C16—H16	120.6
C1—C2—C3	123.80 (18)	C15—C16—H16	120.6
C6—C2—C3	118.89 (18)	C16—C17—C18	121.0 (2)
C4—C3—C8	116.89 (17)	C16—C17—H17	119.5
C4—C3—C2	107.82 (17)	C18—C17—H17	119.5
C8—C3—C2	112.82 (17)	C19—C18—C17	120.0 (2)
C4—C3—C7	108.55 (16)	C19—C18—H18	120.0
C8—C3—C7	100.89 (16)	C17—C18—H18	120.0
C2—C3—C7	109.49 (16)	C18—C19—C20	120.0 (2)
C5—C4—C14	119.68 (19)	C18—C19—H19	120.0
C5—C4—C3	123.41 (18)	C20—C19—H19	120.0
C14—C4—C3	116.85 (18)	C15—C20—C19	118.8 (2)
C4—C5—O1	123.57 (19)	C15—C20—C5	116.64 (19)
C4—C5—C20	122.36 (19)	C19—C20—C5	124.40 (19)
O1—C5—C20	114.06 (18)	O5—C21—N4	125.8 (3)
N2—C6—C2	178.5 (2)	O5—C21—H21	117.1
O2—C7—N3	127.16 (19)	N4—C21—H21	117.1
O2—C7—C3	124.40 (19)	N4—C22—H22A	109.5
N3—C7—C3	108.38 (17)	N4—C22—H22B	109.5
C9—C8—C13	118.63 (19)	H22A—C22—H22B	109.5
C9—C8—C3	132.46 (19)	N4—C22—H22C	109.5
C13—C8—C3	108.84 (18)	H22A—C22—H22C	109.5
C8—C9—C10	120.6 (2)	H22B—C22—H22C	109.5
C8—C9—Br1	120.17 (16)	N4—C23—H23A	109.5
C10—C9—Br1	119.23 (16)	N4—C23—H23B	109.5
C11—C10—C9	119.3 (2)	H23A—C23—H23B	109.5
C11—C10—H10	120.3	N4—C23—H23C	109.5
C9—C10—H10	120.3	H23A—C23—H23C	109.5
C10—C11—C12	121.7 (2)	H23B—C23—H23C	109.5
C10—C11—H11	119.2		
			
C5—O1—C1—N1	−175.77 (17)	C13—C8—C9—Br1	178.87 (15)
C5—O1—C1—C2	4.4 (3)	C3—C8—C9—Br1	−4.6 (3)
N1—C1—C2—C6	6.6 (3)	C8—C9—C10—C11	−0.4 (3)
O1—C1—C2—C6	−173.60 (18)	Br1—C9—C10—C11	−179.33 (16)
N1—C1—C2—C3	−172.6 (2)	C9—C10—C11—C12	0.4 (3)
O1—C1—C2—C3	7.1 (3)	C10—C11—C12—C13	0.1 (3)
C1—C2—C3—C4	−11.5 (3)	C11—C12—C13—C8	−0.6 (3)
C6—C2—C3—C4	169.26 (18)	C11—C12—C13—N3	−179.9 (2)
C1—C2—C3—C8	119.1 (2)	C9—C8—C13—C12	0.5 (3)
C6—C2—C3—C8	−60.1 (2)	C3—C8—C13—C12	−176.78 (19)
C1—C2—C3—C7	−129.4 (2)	C9—C8—C13—N3	179.94 (18)
C6—C2—C3—C7	51.3 (2)	C3—C8—C13—N3	2.6 (2)
C8—C3—C4—C5	−122.6 (2)	C7—N3—C13—C12	−178.8 (2)
C2—C3—C4—C5	5.7 (3)	C7—N3—C13—C8	1.8 (2)
C7—C3—C4—C5	124.3 (2)	C15—O3—C14—O4	178.80 (19)
C8—C3—C4—C14	60.4 (2)	C15—O3—C14—C4	−0.4 (3)
C2—C3—C4—C14	−171.33 (17)	C5—C4—C14—O4	178.0 (2)
C7—C3—C4—C14	−52.8 (2)	C3—C4—C14—O4	−4.8 (3)
C14—C4—C5—O1	−178.31 (18)	C5—C4—C14—O3	−2.9 (3)
C3—C4—C5—O1	4.7 (3)	C3—C4—C14—O3	174.33 (17)
C14—C4—C5—C20	3.2 (3)	C14—O3—C15—C16	−175.4 (2)
C3—C4—C5—C20	−173.79 (19)	C14—O3—C15—C20	3.4 (3)
C1—O1—C5—C4	−10.4 (3)	O3—C15—C16—C17	178.0 (2)
C1—O1—C5—C20	168.17 (17)	C20—C15—C16—C17	−0.8 (3)
C13—N3—C7—O2	177.2 (2)	C15—C16—C17—C18	1.3 (4)
C13—N3—C7—C3	−5.3 (2)	C16—C17—C18—C19	−0.8 (4)
C4—C3—C7—O2	−52.7 (3)	C17—C18—C19—C20	−0.2 (3)
C8—C3—C7—O2	−176.1 (2)	O3—C15—C20—C19	−178.96 (19)
C2—C3—C7—O2	64.8 (3)	C16—C15—C20—C19	−0.2 (3)
C4—C3—C7—N3	129.72 (18)	O3—C15—C20—C5	−3.0 (3)
C8—C3—C7—N3	6.3 (2)	C16—C15—C20—C5	175.7 (2)
C2—C3—C7—N3	−112.82 (19)	C18—C19—C20—C15	0.7 (3)
C4—C3—C8—C9	60.5 (3)	C18—C19—C20—C5	−174.9 (2)
C2—C3—C8—C9	−65.3 (3)	C4—C5—C20—C15	−0.3 (3)
C7—C3—C8—C9	177.9 (2)	O1—C5—C20—C15	−178.91 (18)
C4—C3—C8—C13	−122.70 (19)	C4—C5—C20—C19	175.4 (2)
C2—C3—C8—C13	111.45 (19)	O1—C5—C20—C19	−3.2 (3)
C7—C3—C8—C13	−5.3 (2)	C23—N4—C21—O5	−0.7 (4)
C13—C8—C9—C10	0.0 (3)	C22—N4—C21—O5	179.4 (2)
C3—C8—C9—C10	176.5 (2)		

### Hydrogen-bond geometry (Å, °)

**Table:**

*D*—H···*A*	*D*—H	H···*A*	*D*···*A*	*D*—H···*A*
N1—H1A···O2^i^	0.89 (3)	2.02 (3)	2.891 (2)	165 (2)
N1—H1B···N2^ii^	0.84 (3)	2.27 (3)	3.090 (3)	166 (2)
N3—H3···O5^iii^	0.88	1.93	2.785 (2)	163
C11—H11···O2^iv^	0.95	2.54	3.462 (3)	165
C19—H19···O4^i^	0.95	2.50	3.173 (3)	128
C22—H22A···N2^v^	0.98	2.48	3.443 (3)	166

Symmetry codes: (i) *x*, −*y*+1/2, *z*−1/2; (ii) −*x*+2, −*y*, −*z*+1; (iii) *x*, −*y*+1/2, *z*+1/2; (iv) *x*, *y*−1, *z*; (v) *x*, *y*+1, *z*.
